# Implantation and Recovery of Long-Term Archival Transceivers in a Migratory Shark with High Site Fidelity

**DOI:** 10.1371/journal.pone.0148617

**Published:** 2016-02-05

**Authors:** Danielle E. Haulsee, Dewayne A. Fox, Matthew W. Breece, Tonya M. Clauss, Matthew J. Oliver

**Affiliations:** 1 College of Earth, Ocean and the Environment, University of Delaware, Lewes, DE, United States of America; 2 Department of Agriculture and Natural Resources, Delaware State University, Dover, DE, United States of America; 3 Georgia Aquarium, Atlanta, GA, United States of America; Pacific Northwest National Laboratory, UNITED STATES

## Abstract

We developed a long-term tagging method that can be used to understand species assemblages and social groupings associated with large marine fishes such as the Sand Tiger shark *Carcharias taurus*. We deployed internally implanted archival VEMCO Mobile Transceivers (VMTs; VEMCO Ltd. Nova Scotia, Canada) in 20 adult Sand Tigers, of which two tags were successfully recovered (10%). The recovered VMTs recorded 29,646 and 44,210 detections of telemetered animals respectively. To our knowledge, this is the first study to demonstrate a method for long-term (~ 1 year) archival acoustic transceiver tag implantation, retention, and recovery in a highly migratory marine fish. Results show low presumed mortality (n = 1, 5%), high VMT retention, and that non-lethal recovery after almost a year at liberty can be achieved for archival acoustic transceivers. This method can be applied to study the social interactions and behavioral ecology of large marine fishes.

## Introduction

Acoustic telemetry is commonly used to study the movements, migrations, habitat associations, and more recently survival of marine fishes [1–4]. Measuring social structure and networks of aquatic species is problematic because behavioral observations can be difficult to obtain in the ocean. Therefore, the inter- and intraspecific groups that are formed by marine species are generally poorly understood [5]. Researchers have attempted to observe these social groups using photo identification [6,7], visual observations [8], mark and recapture [9], and standard acoustic telemetry (active tracking: [10], passive tracking: [11]).

An alternative method of observing social structure in marine animals is to use archival tags that record detections of nearby telemetered animals. The miniaturization of acoustic receivers makes it possible for researchers to use marine animals as bio-loggers or mobile telemetry assets [5,12]. However, most archival telemetry receivers must be recovered to retrieve the acoustic detection information. Pinnipeds have proven to be useful mobile telemetry assets because they often travel far distances, are relatively social, and reliably return to haul-out areas allowing researchers to easily locate and retrieve archival tags [13–15]. Archival acoustic telemetry transceivers allow researchers to directly observe interactions between pinnipeds and other species on long time scales and over large geographic ranges. Similarly, internal archival proximity sensors were successfully deployed and recovered from Lemon sharks *Negaprion brevirostris* at liberty for two weeks, however these sharks were selected due to their high degree of natal homing behavior in shallow water and ease of tag recovery [12]. External archival acoustic transceivers have also successfully been deployed on Tiger Sharks *Galeocerdo cuvier*, where they were recaptured after 20–132 days at liberty [5]. However, external tag shedding in fish can be problematic, especially for large transmitters coupled with long deployments [16–18]. Bio-fouling on external tags can also increase shedding rates or tag failure, as well cause reoccurring damage to the tagged animal and cause animosity between stakeholders (anglers, tourists, etc.) and scientist [19].

Here, we present a method for internal attachment and recovery of archival acoustic transceivers in the coelomic cavity of a coastal shark, the Sand Tiger *Carcharias taurus*. Sand Tigers are a highly migratory shark found in coastal oceans worldwide, including along the Eastern Coast of the USA, and are globally listed as “Vulnerable” on the IUCN Red List [20] with two sub-populations deemed critically endangered [21,22]. This species commonly forms large aggregations [23], making it a good candidate for understanding social structures using data recovered from internally implanted VMTs. We deployed internally implanted VMTs in 20 adult Sand Tigers, of which two tags were successfully recovered (10%) after almost one year at liberty. The surgically recovered VMTs recorded 29,646 and 44,210 detections of telemetered animals respectively. Both sharks resumed active movements after VMT recovery.

## Methods

### Ethics Statement

All Sand Tigers were caught, handled, and released with the permission of the Delaware Department of Natural Resources and Environmental Control (DNREC; 2012-021F), and with approval from the University of Delaware IACUC (1259-2014-0).

### Transceiver Preparation

We used archival VEMCO Mobile Transceivers (VMTs; VEMCO LTD. Nova Scotia, Canada), measuring 35 by 180mm and weighing 280g in air and 122g in seawater. The VMT has a depth rating of 1000m and transmits coded acoustic signals at 69kHz (161dB). All VMTs were programmed to transmit on average every 90s (range 60–120s) with a 100% receiver duty cycle (receiver always on). This transmission and receiver configuration allowed for approximately one year estimated battery life. Before implantation, VMTs were coated with Platinum Silicone Elastomer (Factor II, Inc., Lakeside, AZ) to reduce the chance of rejection [24].

### Shark Capture

Sand Tigers were captured between 5 July and 24 September 2012 using bottom longline techniques adapted from McCandless et al. [25]. Mainlines were approximately 305m of 0.64cm braided nylon, with barbless Mustad 12/0 circle hooks placed approximately every 12-13m. Each hook was baited with half of an Atlantic Menhaden *Brevoortia tyrannus*. Elapsed time from deployment until removal (combined soak and processing time) averaged 3.02 ± 0.94hr. Sex, fork length (FL), total length (TL) and general condition were recorded for every Sand Tiger captured. All Sand Tigers were tagged with a National Marine Fisheries Service (NMFS) ‘M-type’ shark tag at the base of their first dorsal fin.

A subset of 20 adult Sand Tigers (male TL > 190cm, female TL > 220cm; [26]) were implanted with VMTs (Table 1). Upon capture, each shark was secured alongside the boat, and placed into tonic immobility by orienting the shark ventral side up [27], with the support of a canvas strap (~10cm wide), or a canvas sling. All sharks remained immersed with water flowing over the gills. Oxytetracycline (OTC Bio-Mycin, 10mg/kg of bodyweight) was injected with a sterilized syringe subcutaneously in the area under the dorsal fin, for protection against infection and to mark the vertebrae for aging at a later date. Bodyweight was estimated from FL according to conversion equations provided by Goldman et al. [26].

**Table 1:** Tagging and recapture information for adult Sand Tigers *Carcharias taurus* implanted with VEMCO Mobile Transceivers (VMTs).

Shark ID	Original Capture Date	Fork Length (Total Length) cm	Sex	Recapture Date	Days at Liberty
Shark 1	19-Aug-12	206 (243)	male	-----	-----
Shark 2	20-Aug-12	211 (252)	female	-----	-----
Shark 3	20-Aug-12	225 (261)	female	-----	-----
Shark 4	21-Aug-12	225 (263)	female	-----	-----
Shark 5	21-Aug-12	226 (267)	female	-----	-----
Shark 6	21-Aug-12	233 (-----)	female	-----	-----
Shark 7	21-Aug-12	226 (272)	female	-----	-----
Shark 8	23-Aug-12	219 (260)	female	-----	-----
Shark 9	23-Aug-12	224 (260)	female	-----	-----
Shark 10	24-Aug-12	208 (251)	male	-----	-----
Shark 11	24-Aug-12	194 (235)	male	26-Jul-13	336
Shark 12	24-Aug-12	187 (222)	female	-----	-----
Shark 13	24-Aug-12	202 (242)	male	-----	-----
Shark 14	24-Aug-12	198 (233)	male	11-Aug-13	352
Shark 15	30-Aug-12	200 (234)	male	-----	-----
Shark 16	30-Aug-12	230 (269)	female	-----	-----
Shark 17	07-Sep-12	217 (263)	female	-----	-----
Shark 18	07-Sep-12	192 (230)	female	-----	-----
Shark 19	07-Sep-12	210 (255)	female	-----	-----
Shark 20	07-Sep-12	193 (230)	male	-----	-----

### Surgical Procedure: Tag Implantation

**Fig 1:**
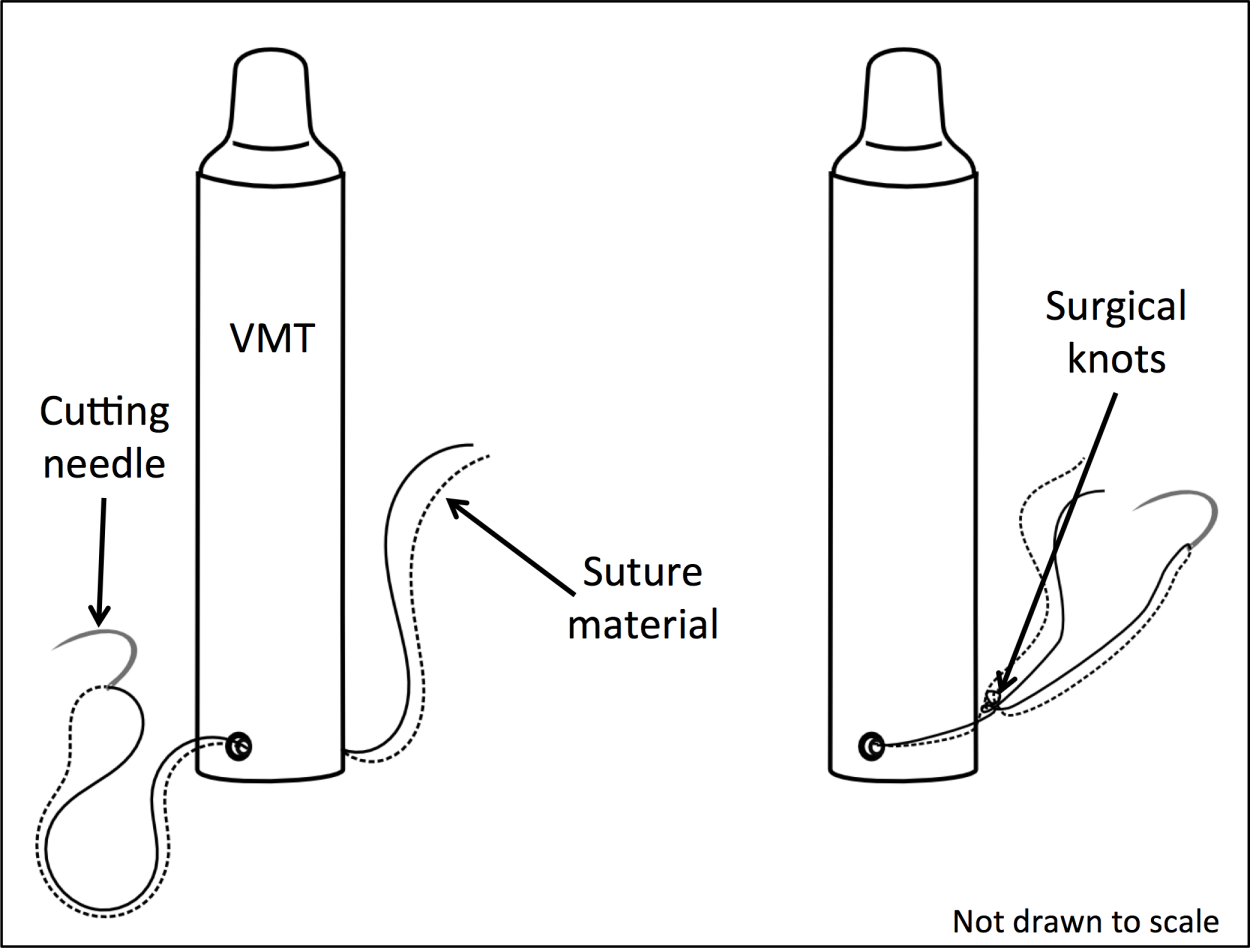
Tag attachment setup. Non-absorbable 18kg nylon monofilament was threaded through the Martin Uterine ½ circle reverse cutting needle (size 6), and attached to the VEMCO Mobile Transceivers (VMTs) by threading the monofilament through a factory-drilled hole on the VMTs. The ends were tied using four surgical knots.

Elastomer coated VMTs were bathed in 91% isopropyl alcohol for sterilization just prior to implantation. In preparation for internal attachment, approximately 2m of sterilized 18kg test nylon monofilament was threaded through a Martin Uterine ½ circle reverse cutting needle (size 6) and secured through the factory drilled holes at the base of the VMT using four surgical knots (Fig 1).

**Fig 2:**
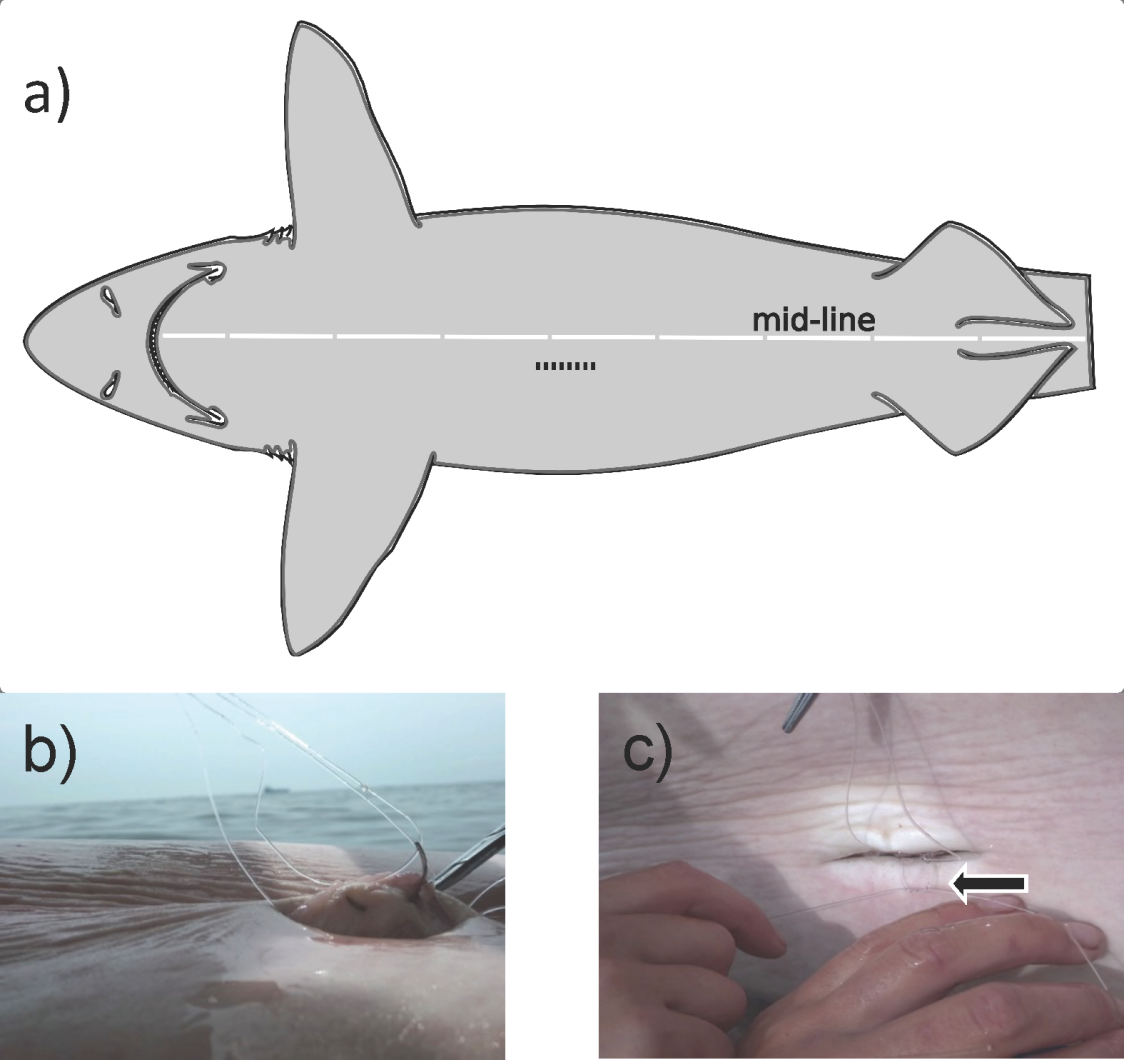
VEMCO Mobile Transceiver (VMT) insertion procedure. a) Location of the ~5cm incision made just off the midline on the ventral side of the animal. The incision went through the body wall and into the peritoneal cavity. Adapted from [28]. b) Martin Uterine ½ circle reverse cutting needle (size 6) inserted through the serosal surface of the peritoneal cavity, but not protruding through the skin. The non-absorbable nylon monofilament was twice looped through this tissue. c) Example of the surgical knots that were tied to secure the 4 loose ends of nylon monofilament looped through the serosal surface of the peritoneal cavity. Four surgical knots were tied with the two pairs of loose ends and ends were trimmed.

Using a sterile carbon-steel scalpel blade (size 11), an ~5cm incision was made just distal to the ventral midline, anterior of the pelvic fins (Fig 2A). The incision was made through the epidermis, dermis and muscle. Forceps were used to pull the body wall away from the viscera in order to protect the organs while making a small cut through the peritoneum, which was enlarged with a pair of blunt scissors. The VMT was then inserted into the coelomic cavity, leaving the attached monofilament and needle outside of the incision. Using a needle driver (16.5cm Olsen Hegar), the monofilament was looped twice through the serous membrane of the peritoneal cavity, on the midline side of the incision (Fig 2B). The needle was cut off of the monofilament, and the tag was secured to the body wall by hand tying four surgical knots with each pair of loose ends. The two sets of loose ends were tied separately to provide better assurance that the VMT would remain anchored to the body wall if one knot failed (Fig 2C). The tails of the monofilament were trimmed and tucked into the coelomic cavity.

With the understanding that telemetered Sand Tigers exhibit high levels of inter-annual site fidelity to Delaware Bay, and that the recapture of tagged individuals was going to depend on acoustically detecting them beyond the life of the VMT, we implanted each VMT shark with VEMCO V16-4H acoustic transmitters programmed to become active just prior to the expiration of the VMT. These smaller transmitters were set with an average transmission interval of 90s from October to June, and an average transmission interval of 60s from July to September to assist in active tracking of the sharks for approximately four years after tagging. These transmitters were also coated in Platinum Silicone Elastomer, and were placed into the coelomic cavity but not secured to the body wall.

The incision was closed using a Ford interlocking continuous suture pattern [29], which reduces the number of knots used, and facilitates appropriate wound closure. Suture material was an absorbable polydioxanone monofilament with a swaged reverse cutting ½ circle needle (ETHICON PDS*II CP-1, size 1). Throughout the procedure, care was taken to hold the shark high enough out of the water to prevent water contamination of the incision site while still maintaining adequate water flow across the gills. Following the procedure, all sharks were returned to ventral recumbency and allowed to recover from tonic immobility before release. All sharks were seemingly stable and actively swam away without complication.

### Tag Monitoring

The Delaware Bay and surrounding coastal waters contained an extensive array of VEMCO VR-2W passive acoustic receivers maintained by Delaware State University that allowed us to monitor the annual emigration and immigration of telemetered Sand Tigers. If a tagged shark returned to the bay within two years after the initial surgery, the shark was considered a survivor of the surgical procedure. For Sand Tigers that did not return to the Delaware Bay, we queried the detection records from arrays in other coastal systems with the cooperation of the Atlantic Cooperative Telemetry (ACT) Network, to assess survival outside of the core monitoring region.

### Manual Tracking and Recapture

The year following tag deployment, active relocation attempts for Sand Tigers carrying VMTs were conducted in the Delaware Bay using a manual tracking acoustic receiver (VEMCO VR100) and both directional (VH110) and omnidirectional (VH165) hydrophones. Once the tag code from a shark carrying a VMT was identified from the omnidirectional hydrophone, the directional hydrophone was used to orient the vessel as close as possible to the shark. When we were within an area of approximately +/- 100m and had established a direction of travel of the shark, a baited bottom longline identical to the one previously described was deployed ahead of the shark’s anticipated travel location.

### Surgical procedure: Tag removal

Once a shark carrying a VMT, positively identified by the manually tracking acoustic receiver and dart-tag number, was recaptured, the VMT was removed. This procedure was similar to the tag insertion procedure; sharks were supported in dorsal recumbency in a tonic state while immersed using a canvas strap. The location of the internally secured VMT was identified from the scar resulting from the original VMT implantation incision. An approximately 8 cm incision was made alongside that scar taking care not to cut the monofilament that secured the VMT to the body wall. When the VMT was located, the monofilament holding it to the body wall was cut and the VMT was removed. Excess monofilament was removed to promote healing. The incision was then closed using an absorbable suture (the same type used in tag implantation procedure) via a modified Ford interlocking or interrupted suture pattern. Before release, both FL and TL were recorded and the old dart tag was removed and replaced with a new one. The V16-4H tag was left in the shark to enable tracking and monitoring survival in subsequent years. Sharks were again given a dose of OTC (10 mg/kg of bodyweight) to help prevent infection.

## Results

### Telemetered Sharks

Between 5 July and 24 September 2012, we set 77 long lines, with 1848 hooks capturing a total of 267 Sand Tigers. We surgically implanted VMTs into 13 adult female and 7 adult male Sand Tigers. Females had an average TL of 256cm (222-272cm), while males were slightly smaller with an average TL of 238cm (230-251cm) (Table 1). The average tag mass burden was <0.004% body weight for all sharks which is much lower than the generally accepted “2%” rule [30], suggesting minimal impact on the Sand Tigers behavior and health.

**Fig 3:**
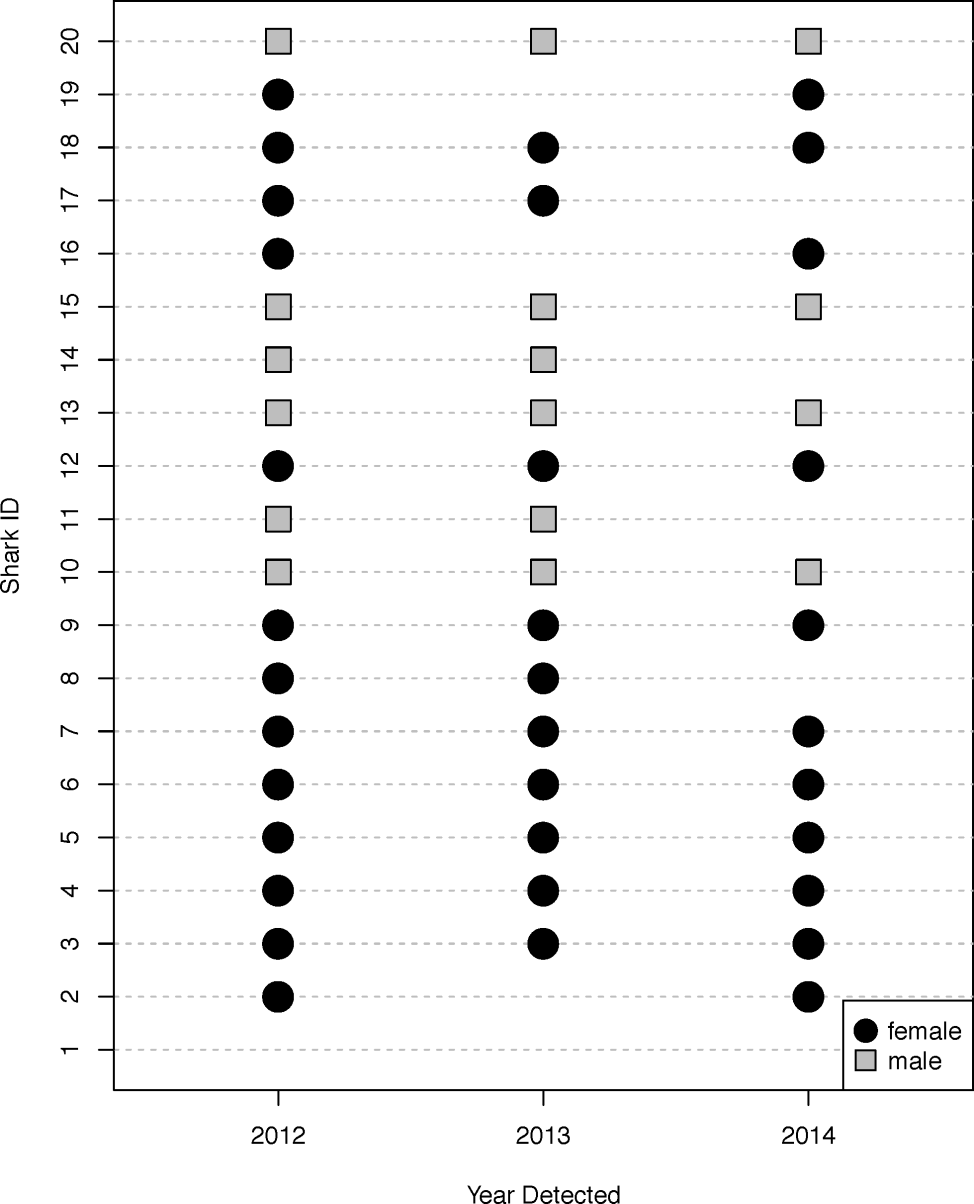
Presence and absence of telemetered sharks. Detections of 20 adult Sand Tigers *Carcharias taurus* outfitted with VEMCO Mobile Transceivers (VMTs) on VEMCO VR-2W moored acoustic receivers located in the Delaware Bay, Delaware USA, the surrounding coastal ocean, and outside receiver arrays along the East Coast United States part of the Atlantic Cooperative Telemetry Network (ACT). All sharks were detected one or two years after surgery except Shark 1.

Of the 20 individuals implanted with VMTs, we detected 19 of the VMTs or V16-4H transmitters on moored passive acoustic receivers within two years after implantation (Fig 3). The only individual never detected after tag implantation was the first male (Shark 1) to receive a VMT, and was presumed to have suffered mortality. The following summer (2013), acoustic detections from the passive receiver array in the Delaware Bay, indicated that 10 of the 19 presumed alive sharks had returned. However, an additional 6 Sand Tigers carrying VMTs were detected by acoustic receivers that were part of the Atlantic Cooperative Telemetry (ACT) Network (outside of the Delaware Bay) at some point throughout the following year. In 2014, 15 of the 19 presumed alive sharks were detected in the Delaware Bay or on other receivers within the ACT Network (Fig 3). No shark, other than Shark 1, went more than one year without being detected by receivers in the Delaware Bay or by receivers that were part of the ACT Network (Fig 3).

### Recapture Events

**Fig 4:**
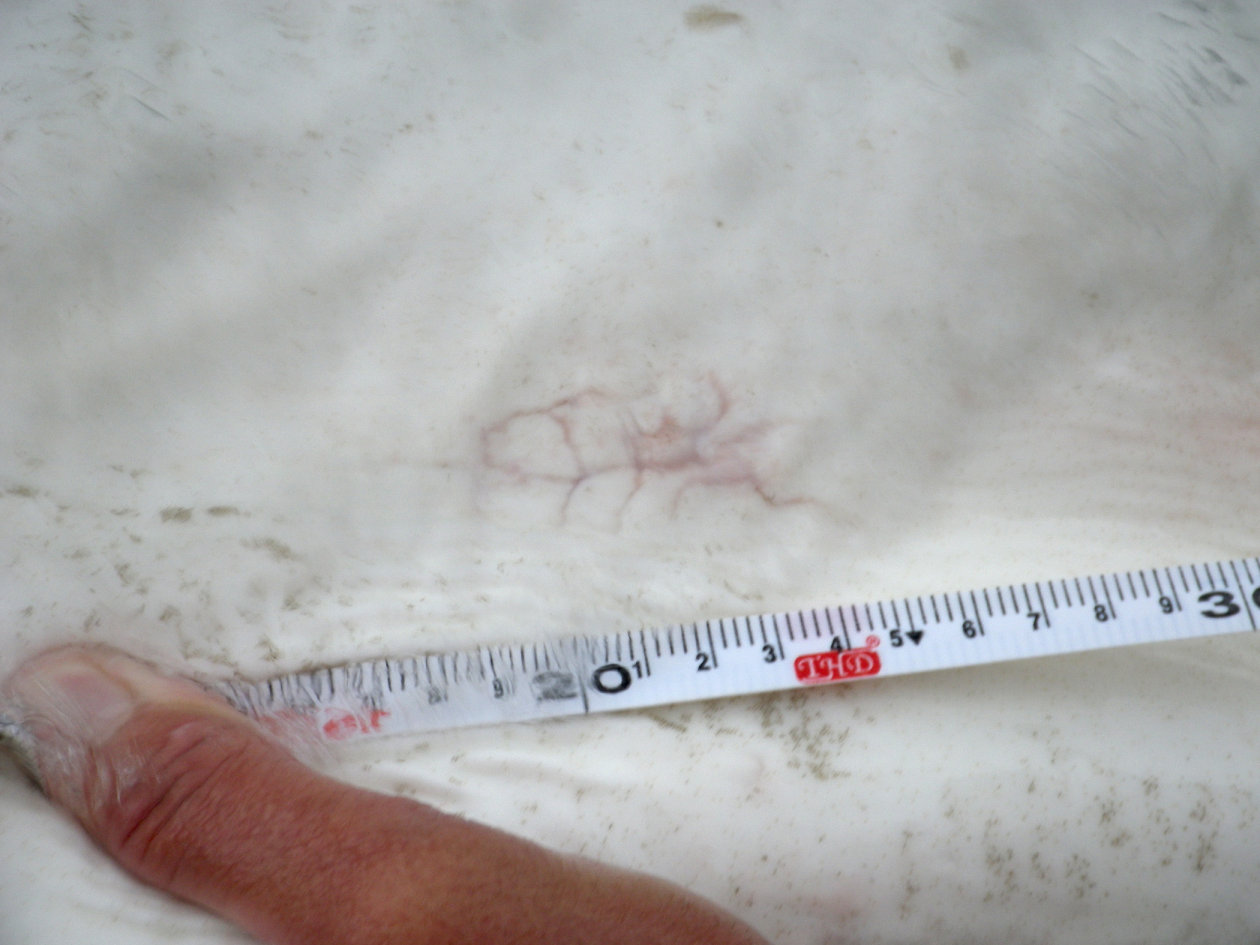
The healed scar from initial tag implantation. The surgical scar was just off the midline of Shark 14 when it was recaptured and the implanted VEMCO Mobile Transceiver (VMT) was recovered. The shark had been at liberty for 352 days after initial tagging. The shark was tagged and recaptured in the Delaware Bay, Delaware USA.

We successfully recaptured two Sand Tigers (Shark 11, 26 July 2013 and Shark 14, 11 August 2013) implanted with VMTs after 336 and 352 days at liberty respectively, through the deployment of 68 longline sets and 1632 hooks. Both individuals retained their VMTs, with fully healed incision sites showing no evidence of infection (Fig 4). The VMT was still internally tethered to the body wall in Shark 11, with no evidence of scar tissue encapsulation on either the transceiver or suture material used for attachment to the body wall. To remove the transceiver, we attempted to clamp onto the knots attaching the monofilament to the VMT, however when the monofilament was compressed, it parted and we had to recover the VMT by hand. In Shark 14, the monofilament was no longer intact. However, the VMT was easily located and retrieved, with no tissue encapsulation.

The detection record from the VMT in Shark 11 was downloaded using a VMT Optical Reader (VEMCO Ltd.). The VMT in Shark 14 was recovered after the battery had expired, but the data were recovered by VEMCO Ltd. from the non-volatile flash memory. The data recovered from the VMTs included the detection date and time, and the tag code detected. The VMT in Shark 11 recorded 29,646 raw detections of animals carrying compatible VEMCO acoustic transmitters, while the VMT in Shark 14 recorded 44,210 raw detections.

Both Shark 11 and Shark 14 were detected on receivers within the Delaware Bay for 25–44 days respectively after the tag recovery and showed a return to active movement within the bay suggesting that both individuals survived the recapture and recovery process.

## Discussion

We consider a 10% VMT recovery rate a success. Through our efforts, we have shown it is possible to recapture telemetered individuals, and easily recover internally tethered tags. In addition, it does not appear that the behavior of the Sand Tigers was affected by the presence of the internally attached VMT. All but one telemetered shark were documented emigrating from the Delaware Bay by October as expected, with the vast majority returning in subsequent years. The sharks that did not return after one year (Sharks 2, 16 and 19) were females (except Shark 1, possible mortality or tag rejection), and there has been anecdotal evidence that females do not return to the bay with the same regularity as males. Sexual segregation in migration patterns has been shown using satellite tags on Sand Tigers tagged in the Delaware Bay [31], which supports the observation of females not returning the first year. In addition, studies have suggested a biennial reproductive cycle for female Sand Tigers in the NW [32] and SW [33] Atlantic Oceans, which may be related to the irregularity of the females returning to the Delaware Bay. All females that did not return the first year after implantation did return the second year, confirming their survival.

We did not observe transmitter encapsulation or rejection in either of the two sharks we recaptured. The lack of encapsulation observed in the sharks recovered suggests that the Platinum Silicone Elastomer used in our study may have reduced internal irritation in Sand Tigers, however no control was used for comparison. Reducing tissue encapsulation around the tag is important for ensuring that the tag can be recovered from an animal with minimal damage, thus increasing the likelihood of survival of the animal. In addition, the retention of the larger transceivers, compared to traditional acoustic transmitters, suggests that the elastomer coating may have reduced tag rejection, although further study is needed to definitively attribute the coating to tag retention.

The monofilament used to anchor the VMT tags to the body wall in our study did not perform as well as intended. After a year at liberty, the monofilament in Shark 11 broke as soon as it was handled, and in Shark 14 the monofilament broke either while the shark was at liberty or was cut while the surgeon made the second incision. It is difficult to determine if heavier nylon monofilament would have maintained flexibility better than the 18 kg monofilament used in our study. In future studies, researchers may consider using non-absorbable monofilament made of other material (polypropylene, polyamide, etc.). While braided suture material can be easier to handle and can decrease time of surgery, it is also more irritating to tissue and silk sutures are less likely to stay intact and therefore not recommended for this application [34].

Our preliminary results, from detections on moored acoustic receivers, indicates a possible mortality rate of 5% for Sand Tigers implanted with a VMT, although the cause of the absence of Shark 1 was not confirmed. While few telemetry studies involving sharks report a mortality rate, some studies have shown post-release survival rates of 90–100% [35,36]. A study by Cooke et al. [34] found that post-surgery mortality in juvenile Largemouth Bass *Micropterus salmoides* was much higher for individuals tagged by a novice surgeon because of the longer surgery times, larger incisions, and misplacement of sutures. While the surgeon in our study had experience implanting transmitters in numerous sharks, this study utilized a new method of implantation, and a learning curve was associated with the implantation procedure, especially for Shark 1. In addition, the Mid-Atlantic region has seen an increase in recreational anglers targeting Sand Tigers [37], which are considered a Species of Concern by the National Marine Fisheries Service requiring anglers to immediately release all incidentally landed individuals. Mortality from recreational angling for Sand Tigers has been documented in different systems worldwide [38–41]. A recently completed study examining the influence of post-release survival of recreationally landed Sand Tigers near the Delaware Bay estimated a 6% mortality rate [39]. As such, we cannot discount the possibility that Shark 1 was recaptured in the coastal ocean shortly after the implantation surgery and suffered mortality thereafter. Finally, it is possible the shark rejected both acoustic tags (VMT and V16-4H) or permanently emigrated from the system.

The high site fidelity of the Sand Tigers captured in the Delaware Bay was critical to the success of this project. Studies using archival tags to study species interactions and associations among individuals in a marine species have previously relied on the animals to haul-out [13–15], to be confined in a pen [42], or to be relatively fixed to a small area like a nursery habitat [12]. Here we show that it is possible to safely recover internal tags, independent of fisheries, from a highly mobile species that exhibits homing behavior. Several species of interest also exhibit site fidelity and could be candidates for future studies using this tagging technique. For example, White Sharks *Carcharodon carcharias* have been observed making predictable migrations between hot spots in the Pacific Ocean [43,44]. In addition, Bonnethead Sharks *Spyrna tiburo* have been documented with perhaps the highest recorded degree of site fidelity of any migratory shark [45], however a smaller transceiver may be necessary for this species. Finally, elasmobranch species that are tied to specific habitat types and may not make large-scale movements but also exhibit interesting social structure such as Nurse Sharks *Ginglymostoma cirratum* [46,47] and Lemon Sharks [12] may be appropriate for this type of research, and tag recovery may be less challenging. The method presented in this paper could be a useful approach for researchers studying social behavior in a variety of large marine fishes. The continued development of smaller tags and additional acoustic transceiver technological advances may open up additional opportunities for other species of interest. Advances such as these are key to understanding the social interactions and behavioral ecology of these species.
